# Resistance Evaluation for Native Potato Accessions against Late Blight Disease and Potato Cyst Nematodes by Molecular Markers and Phenotypic Screening in India

**DOI:** 10.3390/life13010033

**Published:** 2022-12-23

**Authors:** Dalamu, Jagesh Kumar Tiwari, Aarti Bairwa, Nisha Bhatia, Rasna Zinta, Nimisha Kaushal, Vinod Kumar, Ashwani K. Sharma, Sanjeev Sharma, Babita Choudhary, Satish Kumar Luthra, Tanuja Buckseth, Rajesh K. Singh, Ajay K. Thakur, Manoj Kumar, Devendra Kumar

**Affiliations:** 1 Central Potato Research Institute, Shimla 171001, Himachal Pradesh, India; 2 School of Biotechnology, Shoolini University, Solan 173229, Himachal Pradesh, India; 3 School of Bioengineering and Biosciences, Lovely Professional University, Phagwara 144001, Punjab, India; 4 ICAR-Central Potato Research Institute, Regional Station, Modipuram, Meerut 250110, Uttar Pradesh, India

**Keywords:** late blight, potato cyst nematode, resistance, molecular marker, phenotype screening

## Abstract

The potato originated in southern Peru and north-western Bolivia (South America). However, native accessions have also been cultivated in India for many years. Late blight, caused by the fungus *Phytophthora infestans*, is the most devastating potato disease, while potato cyst nematode (*Globodera* spp.) (PCN) is another economically significant quarantine-requiring pest in India. In this study, we have generated a new Indian native collection of 94 potato accessions collected from different parts India. These accessions were screened against late blight and potato cyst nematode resistance by using gene-based molecular markers and phenotypic screening methods. Marker assisted selection using *R1* gene-specific marker CosA_210_ revealed a late blight resistance gene in 11 accessions. PCN resistance bands were found in 3 accessions with marker TG689_141_, 5 accessions with marker 57R_452_, and 1 accession having Gro1-4-1_602_ marker for *G. rostochiensis* (Ro1,4), while 64 accessions amplified marker HC_276_ indicating *G. pallida* (Pa2,3) resistance gene (*GpaV_vrn_ QTL*). On the other hand, phenotypic screening against late blight resistance under natural epiphytic conditions (hot-spot) revealed three accessions with high resistance, while others were resistant (1 accession), moderately resistant (5 accessions), susceptible (29 accessions), and highly susceptible (56 accessions). For *G. rostochiensis* (golden cyst nematode) and *G. pallida* (white cyst nematode) resistance, accessions were grouped into highly resistant (3, 3), resistant (0, 2), moderately resistant (6, 29), susceptible (32, 30), and highly susceptible (53, 30), respectively, for the two PCN species. Collectively, we identified promising accessions with high resistance to late blight (JG-1, Kanpuria Safed, and Rangpuria), and also highly resistant to both *Globodera* species (Garlentic, Jeevan Jyoti, and JG-1). Our findings suggested that these accessions would be useful for late blight and PCN resistance breeding, as well as future molecular studies in potatoes.

## 1. Introduction

Potato (*Solanum tuberosum* L.) is one of the most important non-grain food crops today. The potato originated in the Andes mountain regions of Peru and Bolivia, specifically in the Titicaca lake basin [1]. The genus *Solanum* contains over 2000 species, of which 235 are tuber-bearing potato species. The most recent classification of potato includes *Solanum tuberosum* Gp Andigenum of highland Andean genotypes, *Solanum tuberosum* Gp Chilotanum of lowland Chilean landraces, *Solanum ajanhuiri* (2*x*), *Solanum juzepczukii* (3*x*), and *Solanum curtilobum* (5*x*) [2].

The potato was first introduced in India in the early 17th century. It was brought to India from Europe by Portuguese traders or British missionaries as a temperate, long-day crop that is unsuitable for the country’s subtropical regions [3]. Following that, it spread to different parts of India such as Calicut (Kerala) in 1610, Kolkata (West Bengal) in 1832, Nainital/Dehradun (Uttarakhand) in 1832, Nilgiri (Tamil Nadu) in 1830, and Ooty (Tamil Nadu) in 1848 [4]. Potato farming spread throughout India over the next two and a half centuries after it was introduced as a garden vegetable in Western India [4]. Probably, the first potato introduced to India belonged to *S. tuberosum* Gp. Andigenum. However, these introductions were known by various local names in different dialects/languages, causing confusion about their identification and nomenclature. Based on morphological examinations, the Potato Synonym Committee, National Institute of Agricultural Botany, England identified 16 non-European varieties known as indigenous or native samples or varieties that are the result of survivors of earlier introductions and chance selections in Indian agro-climates and have salient features of tolerance to abiotic stresses such as heat and drought, as well as degenerative viruses [4]. Over time, exploration for such collections was carried out in various parts of the country, and the current repository contains over 100 indigenous accessions conserved in vitro/in vivo at the institute [3].

The potato crop suffers from a variety of abiotic and biotic stresses such as late blight, viruses, potato cyst nematode, bacterial wilt, soil and tuber born diseases and so on. Of these, late blight and potato cyst nematode (PCN) are two of the major biotic stresses impeding potato cultivation in the Himalayan hills of Indian states such as Himachal Pradesh, Uttarakhand, and Jammu and Kashmir. Late blight is the most devastating disease and a worldwide problem, and it is more prevalent in hilly regions but noticed once every two or three years in sub-tropical plains in India as well. Late blight, a fungal infection caused by *Phytophthora infestans* (Mont.) de Bary, causes significant yield losses and necessitates the use of fungicides in all potato growing regions. Late blight affects both foliage and tuber, reducing tuber yield as well as tuber quality [5]. Though initially potato late blight was thought to be a temperate-world disease, the disease is now also prevalent in the subtropical Indian plains, necessitating the development of resistant varieties for these areas [6]. To manage this pathogen due to the emergence of new virulent pathotypes, the search for new chemical formulations as well as resistance sources are important. Compared to fungicidal applications, host resistance is one of the most eco-friendly, cost-effective, sustainable, and consumer-oriented (chemical-free produce) options. Secondly, PCN (*Globodera rostochiensis* and *G. pallida*) species are significant pests that feed on potato roots, causing significant losses of up to 30% [7]. PCN populations coexist in infected soil, warranting PCN resistant genotypes. Both the PCN species are present as mixed populations in all potato-growing areas of the southern hills in India, and a recent investigation has confirmed their presence in the north-western hills of the country. Host resistance is a long-term method of managing late blight and PCN. *S. t.* ssp. *andigena*, *S. vernei*, *S. gourlayi*, *S. sparsipilum*, and *S. spegazzinii* are the most commonly used cultivated and wild species in PCN resistance breeding [7,8]. To identify resistant genotypes against late blight and PCN, phenotypic screening methods have been used extensively. Furthermore, molecular marker-based selection has been deployed to identify resistant genotypes for breeding new cultivars.

The aim of this study was to generate a collection of new native accessions (94 nos.) of potato genotypes from different parts of India. This native collection was characterized by phenotypic screening methods for late blight and PCN resistance. Phenotypic screening for late blight was performed in hotspot natural field conditions, and PCN resistance was tested in soil-inoculation by the root ball technique. Additionally, gene-based molecular markers were also deployed to confirm resistance genes in these accessions, so that promising lines can be used in resistance breeding for developing new cultivars in future.

## 2. Materials and Methods

### 2.1. Plant Materials

A total of 94 new native potato accessions were collected from various parts of India for molecular and phenotypic screening against late blight and PCN resistance. All accessions were conserved in the germplasm repository at Indian Council of Agricultural Research-Central Potato Research Institute, Shimla (31.10° N, 77.17° E, 2276 m above mean sea level), Himachal Pradesh, India, under either in vitro (tissue culture) or in field (tuber) conditions. The in vitro materials are conserved in MS medium [9] supplemented with 40 gL/L sorbitol and 7 gL/L agar [10]. The plants were grown in the field (late blight) and pots (PCN) using a standard set of practices. Plant leaf tissues were collected in triplicate for molecular research.

### 2.2. Molecular Marker Analysis

The DNeasy^®^ Plant Mini Kit was used to isolate DNA from leaf samples from 94 potato accessions following the manufacturer’s instructions (Qiagen, Venlo, Limburg, The Netherlands). The DNA was tested for quality on a 1% agarose gel and quantified with a NanoDrop 2000 Spectrophotometer (Thermo Fisher Scientific, Wilmington, NC, USA). All accessions were screened for late blight and PCN using gene-specific linked molecular markers (Table 1) using the procedures described elsewhere [11]. All primers were synthesized by Eurofins Genomics in Bangalore, India. The polymerase chain reaction (PCR) was set up in a total volume of 10 µL comprised of 100 ng DNA, 1× PCR buffer consisting of 2.5 mM MgCl_2_ and 200 µM dNTP, 1 μL (10 pM) each primer (forward and reverse), 1 U Taq polymerase (Qiagen, Venlo, Limburg, The Netherlands), and Milli-Q water. PCR cycle reaction was executed as follows: 94 °C for 5 min, 35 cycles (94 °C for 45 s, 55–62 °C for 45 s, and 72 °C for 1 min), and 72 °C for 8 min in a Veriti Thermal Cycler (Life Technologies, Carlsbad, CA, USA). Table 1 shows the annealing temperatures of the markers. The PCR products were resolved on 1.5–2.5% agarose gel depending on the size of the marker, and bands were scored using the gel-doc system with the known 100 bp DNA ladder (Alpha Innotech, San Leandro, CA, USA).

### 2.3. Late Blight Resistance Assay

In the summer seasons of 2021 and 2022, all 94 potato accessions were evaluated for late blight resistance in replicated (three), single-row trials under natural (hot-spot) epiphytic conditions at the ICAR-CPRI, Kufri hills (31.09° N, 77.25° E, 2289 m above mean sea level), Shimla, Himachal Pradesh, India including three checks namely Kufri Girdhari (highly resistant), Kufri Himalini (moderately resistant) and Kufri Jyoti (highly susceptible). The area under the disease progress curve (AUDPC) was calculated in percentage-days (%.day) according to the midpoint rule method using the data on disease incidence collected at seven-day intervals between two subsequent readings [20]. Based on the AUDPC value, these accessions were classified as highly resistant/HR (AUDPC: 250), resistant/R (AUDPC: 251–350), moderately resistant/MR (AUDPC: 351–650), susceptible/S (AUDPC: 651–1200), and highly susceptible/HS (AUDPC: >1200) [21]. For disease resistance/susceptibility classification, mean values from both years were pooled for analysis.

### 2.4. Potato Cyst Nematode Assay

All 94 accessions were tested for PCN resistance in the earthen pots using the root ball technique under glasshouse in Kufri conditions using controls SM/11-120 (highly resistant) and Kufri Jyoti (highly susceptible). Five tubers from each accession were planted in 10 cm diameter pots in a glasshouse at 20–22 °C for PCN resistance testing. The experiments were conducted in three replications in two years (2020–2021 and 2021–2022). The planting soil contained 200–250 *Globodera* cysts per 100 mg, resulting in 8000–10,000 eggs and larvae per test tuber. After 60–65 days, when females were visible on the root balls of susceptible control plants, the number of females on the root balls of various accessions was counted. The most desired level of resistance is grade 0–1 clone [22]. The colour of developing females distinguishes the two *Globodera* species. To categorize the data by species, genotypes were graded as highly resistant (0–1 female/root ball), resistant (2–5 female/root ball), moderately resistant (6–20 female/root ball), susceptible (21–50 female/root ball), and highly susceptible (>50 female/root ball), as described elsewhere [22].

## 3. Results

### 3.1. Development of an Indian Native Potato Collection

**Figure 1:**
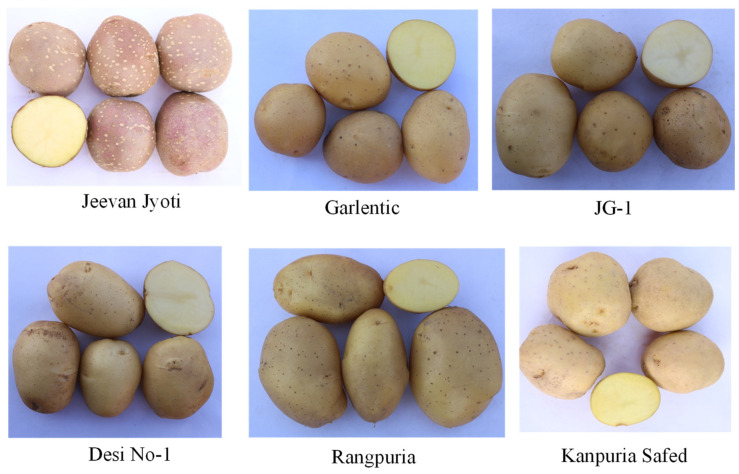
Tuber phenotypes of promising Indian native potato accessions identified based on molecular and phenotypic screening against late blight and potato cyst nematode resistance.

A total of 94 native potato accessions were collected from various parts of India, ranging from plains to hill states, where these potatoes have been grown locally for many years and are maintained through tubers by local growers (Table 2). The list of 94 accessions along with the source are presented in Supplementary File S2. Manipur, Jammu and Kashmir, Assam, Himachal Pradesh, Meghalaya, Sikkim, and West Bengal accounted for 49 accessions, while 43 accessions were collected from Indian plains states such as West Bengal, Uttar Pradesh, Bihar, and Punjab. The origin of the two native accessions Aruconia and PH/C-11 is unknown. The promising resistant accessions identified in this study are depicted in Figure 1.

### 3.2. Marker-Based Screening for Late Blight and PCN Resistance Genes

**Figure 2:**
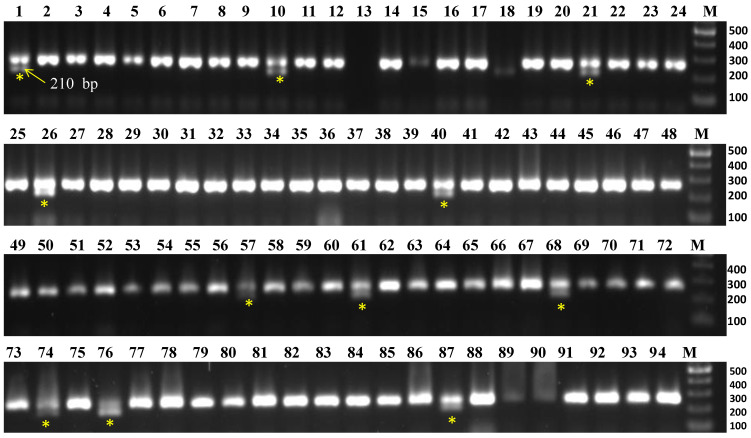
Evaluation of Indian native potato collections for late blight (*Phytophthora infestans*) resistance *R1* gene by using CosA_210_ marker. Yellow asterisk indicates the presence of late blight resistance band (210 bp) in the accessions, viz., NJ-78 (#1), NJ-12 (#10), NJ-47 (#21), VK/JG-2 (#26), Phulwa White (#40), NJ-62 (#57), Var 3797 (#61), Australian White (#68), Aruconia (#74), Assamia Aloo (#76), and Lal Gulab (#87). M = 100 bp DNA ladder.

To screen 94 accessions, three molecular markers, viz., CosA (*R1* gene), R2 (*R2/Rpi-abpt* gene), and R3-1380 (*R3* gene) linked to late blight resistance genes were used. The results are summarized in Table 2. The marker CosA_210_ demonstrated the resistance band (210 bp), indicating the presence of the *R1* resistance gene in 11 accessions namely Aruconia, Assamia Aloo, Australian White, Lal Gulab, NJ-12, NJ-47, NJ-62, NJ-78, Phulwa White, Var 3797, and VK/JG-2 (Figure 2), whereas the remaining accessions lacked this resistance band. Another two markers (R2 and R3-1380) had either no desired fragment amplification or only non-desirable/monomorphic bands, so they were not included in the analysis.

**Figure 3:**
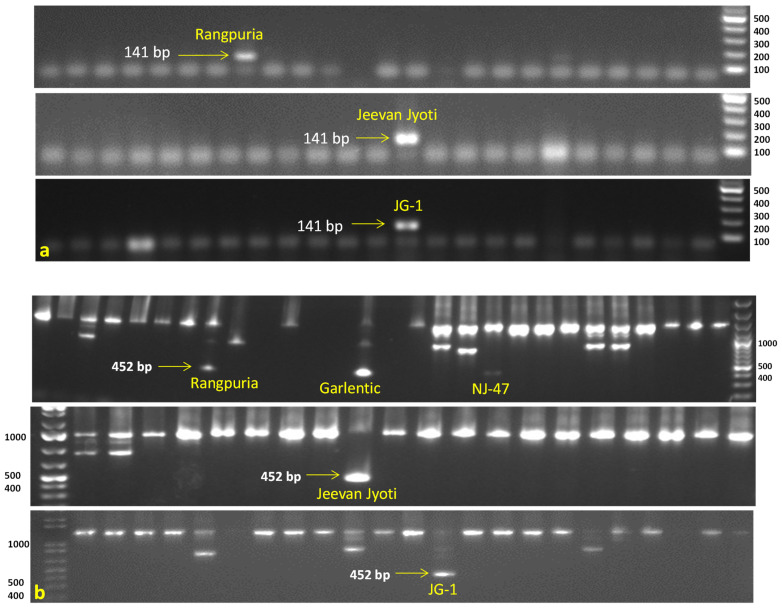
Evaluation of Indian native potato accessions possessing potato cyst nematode (*G. rostochiensis* Ro1, 4) resistance *H1* gene by using molecular markers. (**a**) TG689_141_ marker showed presence of resistance band (141 bp) in three accessions, viz., Rangpuria, Jeevan Jyoti, and JG-1. (**b**) 57R_452_ marker showed presence of resistance band (452 bp) in five accessions, viz., Rangpuria, Garlentic, NJ-47, Jeevan Jyoti, and JG-1. M = 100 bp DNA ladder.

All accessions were also tested for PCN resistance genes using six linked molecular markers against *G. rostochiensis* (Ro1, 4) *viz.*, TG689 (*H1* gene), 57R (*H1* gene), BCH (*H1* gene), and Gro1-4-1 (*Gro1-4* gene), while HC and SPUD1636 markers were associated with *G. pallida* (Pa2/3) resistance genes *GpaV_vrn__QTL* and *Gpa5_QTL*, respectively. The marker TG689_141_ demonstrated the desired amplification of resistance band (141 bp), indicating the presence of the *H1* gene in three accessions (Jeevan Jyoti, JG-1, and Rangpuria) for resistance to *G. rostochiensis* (Ro1, 4). The second marker 57R_452_ revealed the presence of the *G. rostochiensis* resistance gene (*H1* gene) in five accessions namely Rangpuria, Garlentic, NJ-47, Jeevan Jyoti, and JG-1. In contrast, another Gro1-4-1_602_ marker amplified only in one accession JG-1 showed the *Gro1-4* gene conferring resistance to *G. rostochiensis* (Ro1) (Figure 3). Notably, marker HC_276_ was amplified in the majority of acessions (64) indicating the presence of *GpaV_vrn__QTL* which may provide resistance to *G. pallida* (Pa2/3) (Supplementary File S1). Monomorphic or undesirable bands were observed in BCH and SPUD1636 markers, and excluded from the analysis.

### 3.3. Phenotypic Screening for Late Blight and PCN Resistance

**Figure 4:**
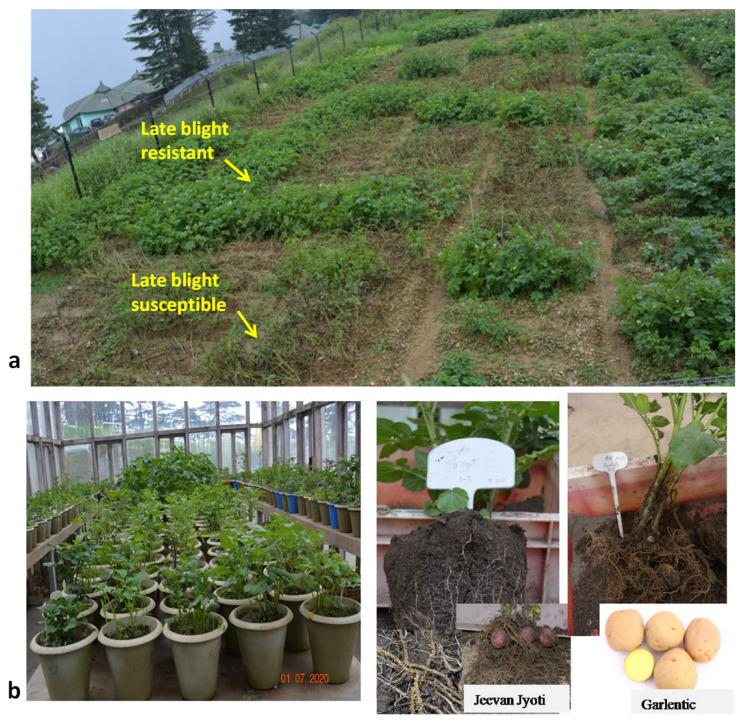
Phenotypic screening of Indian native potato collections under natural epiphytotic field conditions (hot-spot) for late blight resistance (**a**) at Kufri hills, Himachal Pradesh, India, and in the pots for potato cyst nematode resistance through root ball technique (**b**) at Kufri showing highly resistant accessions (e.g., Jeevan Jyoti and Garlentic) to both PCN species.

Phenotypic late blight resistance assays for all 94 accessions were carried out under natural field (hot-spot) conditions in Kufri hills, Shimla, Himachal Pradesh, India for two years (2021 and 2022) during summer seasons (Table 2). A natural field view of late blight resistant/susceptible accessions is depicted in Figure 4. Based on the mean value of two years of data, three native accessions (JG-1, Kanpuria Safed, and Rangpuria) were highly resistant, whereas only one accession (Desi Aloo) was resistant, and five accessions (Australian White, G-4, NJ-47, NJ-62, and VK/JG-2) were moderately resistant to late blight compared to control varieties Kufri Girdhari (highly resistant) and Kufri Jyoti (highly susceptible). The remaining 85 accessions were susceptible or highly susceptible to late blight.

Further, all accessions (94) were also tested twice for PCN resistance in Kufri, Himachal Pradesh in pots using the root ball technique with artificial inoculation of PCN species (Figure 4). PCN results are summarized in Table 2. Three accessions namely Garlentic, Jeevan Jyoti, and JG-1, were found highly resistant to *G. rostochiensis* (Ro1, 4), there were six moderately resistant accessions (JG-25, Lah Sarkari, Lah Saw Khasi, NJ-42, Phulwa Red, Sisapani), and the remaining 85 accessions were either highly susceptible or susceptible. In the case of *G. pallida* (Pa2/3) resistance, the same three accessions (Garlentic, Jeevan Jyoti, and JG-1) were found highly resistant, while only two (Phulwa Red and Ultimus) were resistant, 29 were moderately resistant, and 30 were susceptible as compared to controls (highly resistant: SM/11-120; highly susceptible: Kufri Jyoti). Collectively, Garlentic, Jeevan Jyoti, and JG-1 were identified to be highly resistant to both *Globodera* species, of which JG-1 was highly resistant to late blight also.

## 4. Discussion

The advancements in molecular biology techniques particularly the introduction of various DNA markers have had a significant impact on plant breeding methods. The advantages of DNA-based molecular markers include low cost and high throughput analysis of hundreds of samples at once. Furthermore, it allows for the identification of genes present in the genomes of cultivars with unknown sources of resistance, as well as the early selection of resistant genotypes. Using gene-specific markers, a collection of native potato germplasm was examined for the presence of genes conferring resistance to late blight and PCN [5,7].

Resistance to late blight (*Phytophthora infestans*) is an important goal of potato breeding [8]. To identify genotypes for breeding new cultivars, molecular and phenotypic screening techniques have been used. We found only 11 accessions with the presence of the *S. demissum*-derived *R1* gene-specific marker CosA_210_ [5], of which only four (Australian White, NJ-47, NJ-62, and VK/JG-2) corroborated with phenotypic results (moderately resistant). The remaining seven (Aruconia, Lal Gulab, NJ-12, Assamia Aloo, NJ-78, Phulwa White, and Var3797) amplified CosA_210_ (210 bp) marker but showed phenotypic susceptibility/high susceptibility. This could be due to the *R1* gene being broken down in these accessions, making them susceptible to late blight. Our findings are consistent with earlier work on marker-assisted selection for late blight resistance [9,13]. A study found that the *R1* gene, which was derived from the *S. demissum* background in the 1960s [8], is no longer providing late blight resistance, and hence advocated for the search for genes from wild/semi-cultivated potato species [9]. Based on the phenotypic late blight screening results of a total of nine resistant accessions belonging to different classes, only four were consistent with marker data, i.e., CosA_210_ marker-positive. The remaining five accessions (Desi Aloo, G-4, JG-1, Kanpuria Safed, and Rangpuria) did not amplify this band, indicating the absence of the *R1* gene and the possible presence of different late blight resistance genes. Native collections are widely cultivated in various parts of the country and are likely to have different resistance sources from *Solanum* species that evolved through evolution or chance phenotypic selection [8]. As a result, different species-derived markers using genomics sources are required to discover new genes in these unknown resistance source accessions. These accessions are valuable resources for late blight resistance breeding in potatoes.

Potato cyst nematode particularly *Globodera rostochiensis* resistance genes *H1* and *Gro1-4*, derived from *S. t.* ssp *andigena* CPC1673 and *S. spegazzini*, respectively, were assessed using SCAR markers TG689, 57R, and Gro1-4-1 linked to the respective *R* genes. The *H1* gene provides nearly complete and long-lasting resistance to *G. rostochiensis* (Ro1,4), which is the most common PCN resistance gene in potato cultivars around the world [7]. The TG689_141_ marker was found in three different accessions, viz., Jeevan Jyoti, JG-1, and Rangpuria. Of these, only Jeevan Jyoti, and JG-1 were confirmed as phenotypically highly resistant. Second, the *H1* gene marker 57R_452_ was amplified in five accessions (Rangpuria, Garlentic, NJ-47, Jeevan Jyoti, and JG-1) but only three (Garlentic, Jeevan Jyoti, and JG-1) were phenotypically resistant. Third, only JG-1 accession amplified the Gro1-4-1_602_ marker; it was also confirmed highly resistant by the phenotypic test, indicating the presence of the *Gro1-4* gene conferring resistance to *G. rostochiensis* (Ro1). Overall, only JG-1 possessed all three markers (TG689_141_, 57R_452_, and Gro1-4-1_602_) as well as phenotypically high resistance to *G. rostochiensis* (Ro1,4).

The major loci *GpaV_vrn__QTL* causing resistance to *G. pallida* (Pa2/3) derived from *S. vernei* were applied as the most diagnostic DNA marker HC used worldwide [7]. We identified amplification of the *GpaV_vrn__QTL* specific HC_276_ marker in 64 accessions, which may provide resistance to *G. pallida* (Pa2/3), of which 25 were found resistant in different classes by phenotypic method, but 39 were susceptible. Finally, we identified Garlentic, Jeevan Jyoti, and JG-1 accessions as being highly resistant to both *Globodera* species, and interestingly JG-1 was highly resistant to late blight also. A discrepancy in the detection of this marker was reported in some susceptible forms by Milczarek et al. [23], and TG689 was detected in one susceptible accession previously [24]. Such results may be caused by incomplete dominance of the resistant allele in certain resistant backgrounds, as well as differences in applied marker and pathotype presence. The *H1*, *Gro1-4*, and *S. vernei*-derived genes were identified in common genetic backgrounds [23]. A study showed a poor association between phenotypic resistance and marker presence, while 25% of resistant breeding lines were TG689-negative for the *Ro1* gene [25,26]. The absence of the marker in resistant genotypes could be attributed to alternative sources of resistance such as found in *S. spegazzini* or recombination resulting in the loss of the TG689 marker fragment. The PCN evaluation based on molecular markers is supported by previous findings [27,28]. Our promising accessions have the potential to be a valuable source of PCN resistance in Indian potato breeding programs, demonstrating the importance of this specific accession in broadening PCN resistance. Moreover, the efficiency and reliability of molecular marker assisted selection are well documented in disease resistance potato breeding [29].

In summary, we identified promising accessions highly resistant to late blight (JG-1, Kanpuria Safed, and Rangpuria), and highly resistant both *Globodera* species (Garlentic, Jeevan Jyoti, and JG-1). This research revealed resistant accessions in Indian native collection, which will help to broaden the narrow genetic base of potato breeding against late blight and PCN resistance. Furthermore, it opens opportunities for future discoveries of new resistance genes using modern genomics tools such as transcriptome sequencing and whole genome re-sequencing to identify SNP markers for genomics-assisted breeding.

## Figures and Tables

**Table 1:** Details of molecular markers used in testing a new native collection of 94 potato accession in India for resistance to late blight and potato cyst nematode.

Disease	Marker	Gene	Primer Sequence (5′→3′)	Annealing Temperature (°C)	Size (bp) (Resistant)	References
Late blight (*Phytophthora infestans*)	CosA	*R1*	F: CTCATTCAAAATCAGTTTTGATC R: GAATGTTGAATCTTTTTGTGAAGG	55	210	Gebhardt et al. [12]
	R2	*Rpi-abpt*	F: GCTCCTGATACGATCCATG R: ACGGCTTCTTGAATGAA	55	686	Kim et al. [13]
	R3-1380	*R3*	F: GCTTCCGACATGTATTGATCTCCC R: GGCAGCCACTTCAGCTTCTTACAG	60	1380	Sokolova et al. [14]
Potato cyst nematode *G. rostochiensis/ G. pallida*)	TG689	*H1 * (Ro1, 4)	F: TAAAACTCTTGGTTATAGCCTAT R: CAATAGAATGTGTTGTTTCACCAA	55	141	Galek et al. [15]
	57R	*H1 * (Ro1, 4)	F: TGCCTGCCTCTCCGATTTCT R: GGTTCAGCAAAAGCAAGGACGTG	62	452	Schultz et al. [16]
	BCH	*H1* (Ro1, 4)	F: CATGACATAGTTTGAATTTTGAGTC R: CGTTTGGCGCTGCCGTAAGTT	55	290	Galeket al. [15]
	Gro1-4-1	*Gro1-4 * (Ro1)	F: AAGCCACAACTCTACTGGAG R: GATATAGTACGTAATCATGCC	62	602	Asano et al. [17]
	HC	*GpaV_vrn__QTL* (Pa2/3)	F: ACACCACCTGTTTGATAAAAAACT R: GCCTTACTTCCCTGCTGAAG	60	276	Sattarzadeh et al. [18]
	SPUD1636	*Gpa5_QTL * (Pa2,3)	F: GTGCGCACAGGGTAAAACC R: ACCTTAGCGGATGAAAGCC	60	-	Bryan et al. [19]

Note: Potato cyst nematode pathotypes of *G. rostochiensis* (Ro1, Ro4); *G. pallida* (Pa2/3).

**Table 2:** Evaluation of a new native collection of 94 potato accessions in India based on molecular markers and phenotypic screening methods for resistance to late blight (*Phytophthora infestans*) and potato cyst nematode (*Globodera* spp.).

Sr. No.	Accession	Late Blight		Potato Cyst Nematode					
				*G. rostochiensis *(Ro1, 4)				*G. pallida *(Pa2,3)	
		Marker (CosA_210_)	Phenotype Assay	Marker (TG689_141_)	Marker (57R_452)_	Marker (Gro1-4-1_602_)	Phenotype Assay	Marker (HC_276_)	Phenotype Assay
1.	Aber Chaibi	−	S	−	−	−	S	+	S
2.	AGR/56	−	HS	−	−	−	S	+	HS
3.	Alpha	−	S	−	−	−	S	−	S
4.	Aruconia	+	S	−	−	−	S	+	S
5.	Assamia Aloo	+	HS	−	−	−	HS	+	MR
6.	Australian White	+	MR	−	−	−	HS	+	HS
7.	Badami Aloo	−	S	−	−	−	S	+	S
8.	Bareilly Red	−	HS	−	−	−	HS	−	HS
9.	Beeta	−	S	−	−	−	HS	+	S
10.	Bengal Jyoti	−	HS	−	−	−	HS	−	S
11.	Bhura Aloo	−	HS	−	−	−	HS	+	S
12.	Brondiar Slave	−	S	−	−	−	HS	+	S
13.	Burma Special	−	HS	−	−	−	S	+	MR
14.	C-9-Patna	−	S	−	−	−	HS	−	HS
15.	Champaran Lal	−	HS	−	−	−	HS	+	MR
16.	Clone 1	−	S	−	−	−	HS	+	HS
17.	Dehati Aloo	−	S	−	−	−	HS	+	MR
18.	Deshla Lal	−	HS	−	−	−	S	+	S
19.	Desi Aloo	−	R	−	−	−	HS	−	S
20.	Desi No. 1	−	S	−	−	−	HS	+	S
21.	Desi No. 2	−	HS	−	−	−	HS	−	HS
22.	Dhankri or Tumri	−	HS	−	−	−	HS	+	HS
23.	DRR Blue	−	HS	−	−	−	HS	+	HS
24.	Dwarf Culture	−	HS	−	−	−	HS	−	S
25.	G-4	−	MR	−	−	−	HS	+	S
26.	Garlentic	−	HS	−	+	−	HR	+	HR
27.	Gulabia	−	HS	−	−	−	S	+	MR
28.	Gulmarg Special	−	HS	−	−	−	S	−	MR
29.	Hamraj Hatti	−	HS	−	−	−	S	−	MR
30.	Hyb-3	−	HS	−	−	−	HS	−	MR
31.	Jalandhar	−	S	−	−	−	S	−	S
32.	Jeevan Jyoti	−	HS	+	+	−	HR	+	HR
33.	JG 12	−	S	−	−	−	S	+	MR
34.	JG-1	−	HR	+	+	+	HR	−	HR
35.	JG-22	−	S	−	−	−	HS	+	S
36.	JG-25	−	HS	−	−	−	MR	+	MR
37.	JG-27	−	S	−	−	−	S	+	S
38.	JG-56	−	S	−	−	−	HS	+	HS
39.	K-22	−	HS	−	−	−	HS	−	HS
40.	Kacha Bhutia	−	HS	−	−	−	S	+	S
41.	Kala Aloo	−	HS	−	−	−	S	+	S
42.	Kanpuria Safed	−	HR	−	−	−	HS	+	HS
43.	KP/PC-292	−	HS	−	−	−	HS	+	MR
44.	Lah Arpor	−	HS	−	−	−	HS	−	S
45.	Lah Ipon	−	S	−	−	−	HS	+	HS
46.	Lah Polin	−	S	−	−	−	S	+	MR
47.	Lah Sarkari	−	HS	−	−	−	MR	+	S
48.	Lah Saw	−	HS	−	−	−	HS	−	MR
49.	Lah Saw Khasi	−	HS	−	−	−	MR	−	MR
50.	Lah Saw Smit	−	HS	−	−	−	HS	+	S
51.	Lah Synthiew	−	S	−	−	−	S	+	MR
52.	Lah Tora	−	HS	−	−	−	S	+	S
53.	Lal Ankh	−	HS	−	−	−	HS	−	HS
54.	Lal Gulab	+	S	−	−	−	HS	+	MR
55.	Lal Laukar	−	HS	−	−	−	S	+	MR
56.	Lal Mitti 1	−	HS	−	−	−	HS	−	HS
57.	Lal Mitti 2	−	HS	−	−	−	HS	−	HS
58.	Nainital	−	HS	−	−	−	HS	+	HS
59.	NJ-12	+	S	−	−	−	HS	+	HS
60.	NJ-130	−	S	−	−	−	HS	+	S
61.	NJ-23	−	S	−	−	−	HS	+	HS
62.	NJ-2303	−	S	−	−	−	HS	+	HS
63.	NJ-42	−	HS	−	−	−	MR	+	MR
64.	NJ-47	+	MR	−	+	−	S	+	MR
65.	NJ-56	−	S	−	−	−	S	+	MR
66.	NJ-62	+	MR	−	−	−	S	+	MR
67.	NJ-75	−	HS	−	−	−	HS	−	HS
68.	NJ-78	+	HS	−	−	−	S	+	S
69.	NJ-84	−	HS	−	−	−	S	+	MR
70.	ON-1645	−	HS	−	−	−	HS	−	S
71.	PH/C-11	−	S	−	−	−	S	−	MR
72.	Phulwa Red	−	HS	−	−	−	MR	+	R
73.	Phulwa Red Splashed	−	HS	−	−	−	S	+	MR
74.	Phulwa White	+	HS	−	−	−	HS	+	HS
75.	Pimpernell	−	HS	−	−	−	HS	+	HS
76.	PS-4904	−	HS	−	−	−	HS	−	MR
77.	PSK-76	−	HS	−	−	−	HS	+	HS
78.	R-1	−	HS	−	−	−	S	+	MR
79.	R-2	−	HS	−	−	−	HS	−	S
80.	R-3	−	S	−	−	−	S	+	S
81.	Rangpuria	−	HR	+	+	−	S	+	HS
82.	Red Flesh	−	HS	−	−	−	S	+	MR
83.	Sathoo	−	S	−	−	−	S	+	S
84.	Sisa Pani	−	HS	−	−	−	MR	+	MR
85.	Ultimus	−	HS	−	−	−	HS	−	R
86.	UP to Date	−	S	−	−	−	HS	+	HS
87.	V2-2912	−	S	−	−	−	HS	−	S
88.	Var 3797	+	HS	−	−	−	HS	+	HS
89.	VB-8	−	HS	−	−	−	S	−	S
90.	VK/JG-1	−	HS	−	−	−	HS	+	HS
91.	VK/JG-2	+	MR	−	−	−	HS	+	MR
92.	1001	−	HS	−	−	−	HS	−	HS
93.	1007	−	HS	−	−	−	HS	−	HS
94.	1591/11	−	HS	−	−	−	HS	−	HS

NA: Not available. Phenotypic assay for late blight and PCN resistance is based on mean value of two years data (2021 and 2022). +/− indicates presence/absence of marker associated with the gene. Collection sources are given in Supplementary File S2.

## Data Availability

Not applicable.

## Supplementary Materials

The following supporting information can be downloaded at: https://www.mdpi.com/article/10.3390/life13010033/s1, File S1: DNA amplification profile of two PCN resistance gene linked markers Gro 1-4-1602 and HC276; File S2: A list of 94 native accessions and their collection source in India.
