# Supplementary material for: Resistance Evaluation for Native Potato Accessions against Late Blight Disease and Potato Cyst Nematodes by Molecular Markers and Phenotypic Screening in India
Source: Life (Basel). 2022 Dec 23;13(1):33. doi: 10.3390/life13010033 (PMC9860717; doi:10.3390/life13010033)
Supplement: Supplementary file 1 [file life-13-00033-s001.zip › Suppl. file S1.pdf]

Supplementary file S1. DNA amplification profile of two PCN resistance gene linked markers Gro 1-4-1$_{602}$ and HC$_{276}$

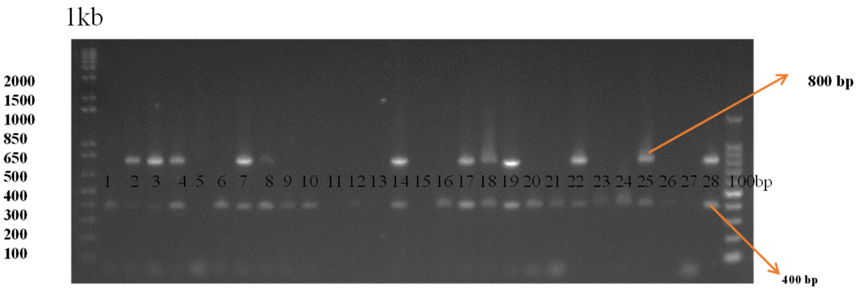

1) NJ-78
2) NJ-2303
3) PS-4904
4) UP to Date
5) Desi Aloo-1
6) 61591/11
7) PH/C-11
8) Rangpuria
9) Phulwa splashed
10) NJ-12
11) NJ-23
12) NJ-75
13) 1007
14) Garlentic
15) Bareilly Red
16) ON-1645
17) Bengal-jyoti
18) Brondar slave
19) JG 12
20) Kanpuria safed
21) NJ-47
22) PR/PC-292
23) K-22
24) Phulwa Red
25) JG-25
26) VK/JG-2
27) Ultimus
28) R-1

Marker Gro 1-4-1

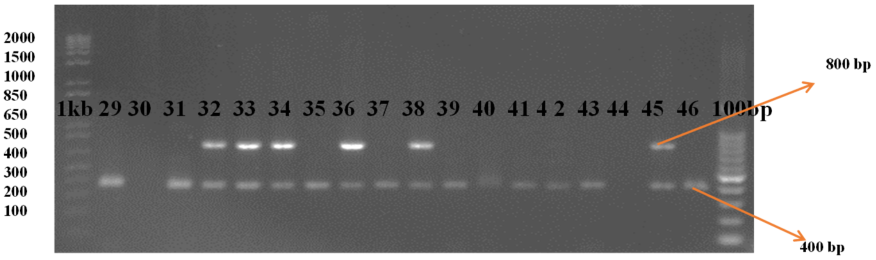

29) Aber- Chaibi
30) Lah sarkar
31) Desi Alu
32) Champaran Lal
33) Nainital
34) Burma special
35) AGR/56
36) Jalandhar
37) Jeevan jyoti
38) Lah Torah
39) NJ-84
40) Phulwa white
41) Lah Arpon
42) Lah Ipon
43) Badami Aloo
44) G-4
45) Lah Smit
46) V2-2912

Marker Gro 1-4-1

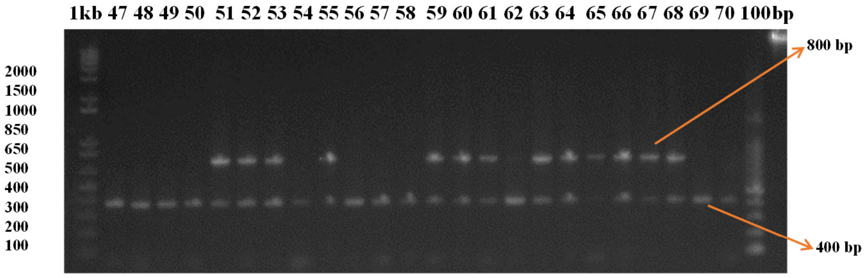

47) Kacha Bhutia
48) Lal Mutti 2
49) Desi No. 2
50) Alpha
51) Lah Saw
52) Clone 1
53) Lal Laukar
54) Lal Ankh
55) Lal Mutti 1
56) Bhura Aloo
57) NJ-62
58) C-9-Patna
59) Pimpernell
60) Dehati Aloo
61) Var 3797
62) Sisapani
63) Sathoo
64) JG-56
65) R-2
66) NJ-42
67) Lah Saw Khasi
68) Australian White
69) Dhankari
70) Red flesh

Marker Gro 1-4-1

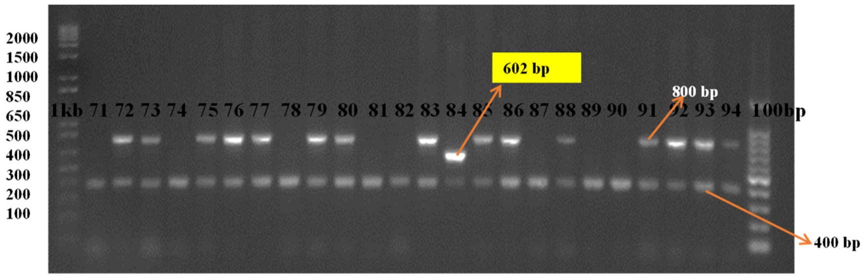

71) 1001
72) Beeta
73) JG-27
74) Aruconia
75) NJ-56
76) Assamia Aloo
77) Lah Synthiew
78) Amaraj Hatti
79) VK/JG-1
80) Lah Polin
81) Gulabia
82) Hyb-3
83) PSK-76
84) JG-1
85) Dwarf culture
86) Gulmarg special
87) Lal agulab
88) R-3
89) Deshla Lal
90) Kala Aloo
91) NJ-130
92) DRR Blue
93) VB-8
94) JG-22

Marker Gro 1-4-1

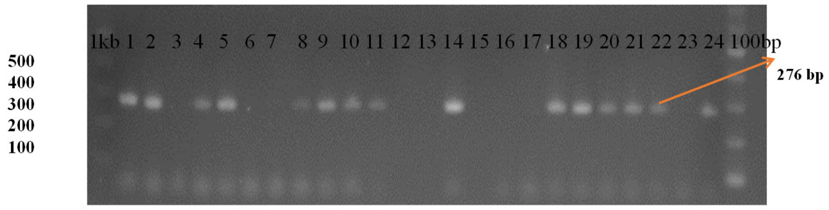

1. NJ-78
2. NJ-2303
3. PS-4904
4. UP to Date
5. Desi Aloo-1
6. 61591/11
7. PH/C-11
8. Rangpuria
9. Phulwa splashed
10. NJ-12
11. NJ-23
12. NJ-75
13. 1007
14. Garlentic
15. Bareilly Red
16. ON-1645
17. Bengal-jyoti
18. Brondar slave
19. JG 12
20. Kanpuria safed
21. NJ-47
22. PR/PC-292
23. K-22
24. Phulwa Red

Marker HC_{276} (PCN)

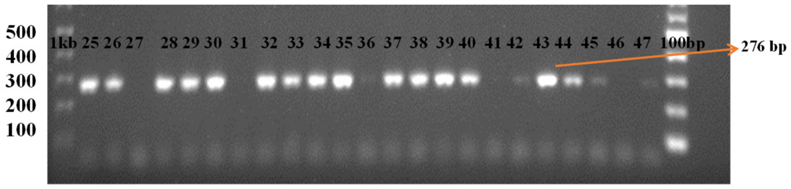

25) JG-25
26) VK/JG-2
27) Ultimus
28) R-1
29) Aber- Chaibi
30) Lah sarkar
31) Desi Alu
32) Champaran Lal
33) Nainital
34) Burma special
35) AGR/56
36) Jalandhar
37) Jeevan jyoti
38) Lah Torah
39) NJ-84
40) Phulwa white
41) Lah Arpon
42) Lah Ipon
43) Badami Aloo
44) G-4
45) Lah Smit
46) V2-2912
47) Kacha Bhutia

Marker HC_{276} (PCN)

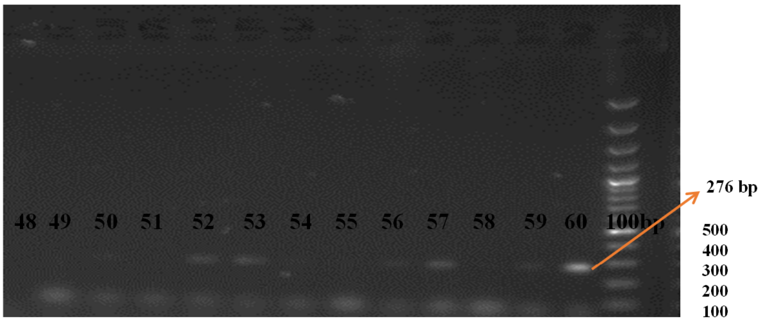

48) Lal Mutti 2
49) Desi No. 2
50) Alpha
51) Lah Saw
52) Clone 1
53) Lal Laukar
54) Lal Ankh
55) Lal Mutti 1
56) Bhura Aloo
57) NJ-62
58) C-9-Patna
59) Pimpernell
60) Dehati Aloo

Marker HC_{276} (PCN)

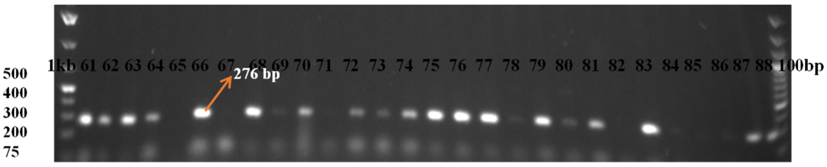

61) Var 3797
62) Sisapani
63) Sathoo
64) JG-56
65) R-2
66) NJ-42
67) Lah Saw Khasi
68) Australian White
69) Dhankari
70) Red flesh
71) 1001
72) Beeta
73) JG-27
74) Aruconia
75) NJ-56
76) Assamia Aloo
77) Lah Synthiew
78) Amaraj Hatti
79) VK/JG-1
80) Lah Polin
81) Gulabia
82) Hyb-3
83) PSK-76
84) JG-1
85) Dwarf culture
86) Gulmarg special
87) Lal agulab
88) R-3

Marker HC_{276} (PCN)

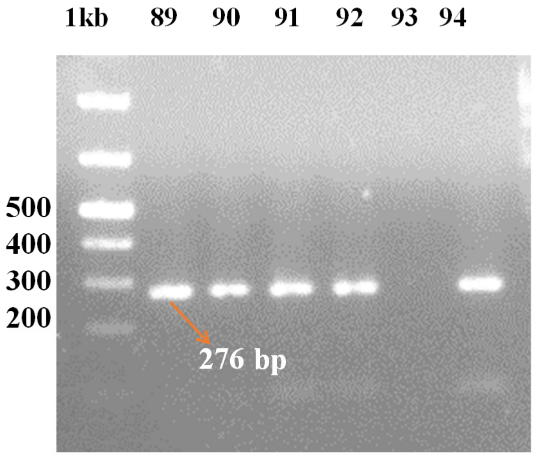

89) Deshla Lal
90) Kala Aloo
91) NJ-130
92) DRR Blue
93) VB-8
94) JG-22

Marker HC_{276} (PCN)
