# Supplementary material for: Resistance Evaluation for Native Potato Accessions against Late Blight Disease and Potato Cyst Nematodes by Molecular Markers and Phenotypic Screening in India
Source: Life (Basel). 2022 Dec 23;13(1):33. doi: 10.3390/life13010033 (PMC9860717; doi:10.3390/life13010033)
Supplement: Supplementary file 1 [file life-13-00033-s001.zip › Suppl. file S2.pdf]

Supplementary file S2. A list of 94 native accessions and their collection source in India

Sr. No.	Accession	Collection source (City, State)	Hill/Plain
1.	Aber Chaibi	Imphal, Manipur	Hill
2.	AGR/56	Kashmir Valley, Jammu & Kashmir	Hill
3.	Alpha	Midnapore, West Bengal	Plain
4.	Aruconia	NA	NA
5.	Assamia Aloo	Jorhat, Assam	Hill
6.	Australian White	Midnapore, West Bengal	Plain
7.	Badami Aloo	Sonapur, Assam	Hill
8.	Bareilly Red	Bareilly, Uttar Pradesh	Plain
9.	Beeta	Gangtok, Sikkim	Hill
10.	Bengal Jyoti	Nalanda, Bihar	Plain
11.	Bhura Aloo	Dholi, Bihar	Plain
12.	Brondiar Slave	Midnapore, West Bengal	Plain
13.	Burma Special	Imphal, Manipur	Hill
14.	C-9-Patna	Patna, Bihar	Plain
15.	Champaran Lal	Champaran, Bihar	Plain
16.	Clone 1	Midnapore, West Bengal	Plain
17.	Dehati Aloo	Patna , Bihar	Plain
18.	Deshla Lal	Nalanda, Bihar	Plain
19.	Desi Aloo	Chamba, Himachal Pradesh	Hill
20.	Desi No. 1	Patna, Bihar	Plain
21.	Desi No. 2	Patna, Bihar	Plain
22.	Dhankri or Tumri	Rampur & Kinnaur, Himachal Pradesh	Hill
23.	DRR Blue	Allahabad, Uttar Pradesh	Plain
24.	Dwarf Culture	Patna, Bihar	Plain
25.	G-4	Farrukhabad, Uttar Pradesh	Plain
26.	Garlentic	Kanpur, Uttar Pradesh	Plain
27.	Gulabia	Dholi, Bihar	Plain
28.	Gulmarg Special	Gulmarg, Jammu & Kashmir	Hill
29.	Hamraj Hatti	Nalanda, Bihar	Plain
30.	Hyb-3	Midnapore, West Bengal	Plain
31.	Jalandhar	Jalandhar, Punjab	Plain
32.	Jeevan Jyoti	Burdwan, West Bengal	Plain
33.	JG 12	Karbi Anglong, Assam	Hill
34.	JG-1	East Khasi hills, Meghalaya	Hill
35.	JG-22	Jammu & Kashmir	Hill
36.	JG-25	Kangra, Himachal Pradesh	Hill
37.	JG-27	Chamba, Himachal Pradesh	Hill
38.	JG-56	Chamba, Himachal Pradesh	Hill
39.	K-22	Midnapore, West Bengal; Farrukhabad, Uttar Pradesh	Plain
40.	Kacha Bhutia	Gangtok, Sikkim	Hill
41.	Kala Aloo	Lahaul-Spiti, Himachal Pradesh	Hill
42.	Kanpuria Safed	Kanpur, Uttar Pradesh	Plain
43.	KP/PC-292	Nakodar, Punjab	Plain
44.	Lah Arpor	Shillong, Meghalaya	Hill
45.	Lah Ipon	Shillong, Meghalaya	Hill
46.	Lah Polin	Shillong, Meghalaya	Hill
47.	Lah Sarkari	Tura, Meghalaya	Hill
48.	Lah Saw	Shillong, Meghalaya	Hill
49.	Lah Saw Khasi	Tura, Meghalaya	Hill
50.	Lah Saw Smit	Shillong, Meghalaya	Hill
51.	Lah Synthiew	Shillong, Meghalaya	Hill
52.	Lah Tora	Shillong, Meghalaya	Hill
53.	Lal Ankh	Jorhat, Assam	Hill
54.	Lal Gulab	Farrukhabad, Uttar Pradesh	Plain
55.	Lal Laukar	Mau, Madhya Pradesh	Plain
56.	Lal Mitti 1	Nalanda, Bihar	Plain
57.	Lal Mitti 2	Nalanda, Bihar	Plain
58.	Nainital	Kokrajhar, Assam	Hill
59.	NJ-12	Kinnaur, Himachal Pradesh	Hill

60.	NJ-130	Kinnaur, Himachal Pradesh	Hill
61.	NJ-23	Kinnaur , Himachal Pradesh	Hill
62.	NJ-2303	Kinnaur, Himachal Pradesh	Hill
63.	NJ-42	Kinnaur, Himachal Pradesh	Hill
64.	NJ-47	Kinnaur, Himachal Pradesh	Hill
65.	NJ-56	Chamba , Himachal Pradesh	Hill
66.	NJ-62	Chamba, Himachal Pradesh	Hill
67.	NJ-75	Chamba , Himachal Pradesh	Hill
68.	NJ-78	Chamba , Himachal Pradesh	Hill
69.	NJ-84	Chamba, Himachal Pradesh	Hill
70.	ON-1645	Birbhum, West Bengal; Khajuri & Farrukhabad, Uttar Pradesh	Plain
71.	PH/C-11	NA	NA
72.	Phulwa Red	Jorhat, Assam	Hill
73.	Phulwa Red Splashed	Jorhat, Assam	Hill
74.	Phulwa White	Jorhat, Assam	Hill
75.	Pimpernell	Midnapore, West Bengal	Plain
76.	PS-4904	Patna, Bihar	Plain
77.	PSK-76	Basti, Uttar Pradesh	Plain
78.	R-1	Samastipur, Bihar	Plain
79.	R-2	Samastipur, Bihar	Plain
80.	R-3	Samastipur, Bihar	Plain
81.	Rangpuria	Cooch Behar, West Bengal	Hill
82.	Red Flesh	Patna, Bihar	Plain
83.	Sathoo	Kinnaur & Chamba, Himachal Pradesh	Hill
84.	Sisa Pani	Patna, Bihar	Plain
85.	Ultimus	Farrukhabad, Uttar Pradesh	Plain
86.	UP to Date	Kinnaur, Himachal Pradesh; Kokrajhar, Assam	Hill
87.	V2-2912	Shimla, Himachal Pradesh	Hill
88.	Var 3797	Allahabad, Uttar Pradesh	Plain
89.	VB-8	Shimla, Himachal Pradesh	Hill
90.	VK/JG-1	Chamba, Himachal Pradesh	Hill
91.	VK/JG-2	Chamba, Himachal Pradesh	Hill
92.	1001	Nakodar, Punjab	Plain
93.	1007	Nakodar, Punjab	Plain
94.	1591/11	Midnapore, West Bengal	Plain
